# Trello

**DOI:** 10.5195/jmla.2016.49

**Published:** 2017-04

**Authors:** Heather A. Johnson

## INTRODUCTION

As libraries continue to evolve in the services that they provide, librarians may be increasingly responsible for managing large projects, planning programs and outreach, and coordinating teams. While some projects can be easily executed by a single person, others may involve a team and several moving parts. In the latter case, a project leader may be responsible for developing a project plan, establishing a timeline, recruiting team members, assigning tasks, managing progress, and apprising sponsors and upper management of the project’s progress.

Managing projects and teams can sometimes seem overwhelming. Fortunately, there are several free project management solutions to assist project leaders in managing their workflow. One of those solutions is Trello, a cloud-based tool that uses the Kanban method of project management. Under the Kanban method, all project-related activities are displayed in a single landscape that is viewable to all members of the project team. With Trello, users can visually organize projects into boards, divide projects into groups, and subdivide groups into tasks. Trello’s user-friendly interface makes it ideal for a wide variety of users, from individuals managing personal projects such as home renovations to organizations managing multiple large projects and teams. To meet users’ needs, Trello offers various levels of service at different price points. This review focuses on the free version.

## MAJOR FEATURES

Trello requires only an Internet connection, eliminating the need for users to install software or enter product keys. Registered users can create an unlimited number of boards and designate one board per project. Users can then assign multiple task groups (lists) to each board and assign subgroups (cards) to each list. Users can create cards by either adding them manually or copying and pasting existing text lists from Microsoft Word or Excel. With the latter method, users have the option to create a card for every line of text or create a single card with multiple lines of text. Cards can be further granulized with to-do lists, which appear only when cards are expanded. Users can also upload attachments and add comments to expanded cards.

**Figure 1:**
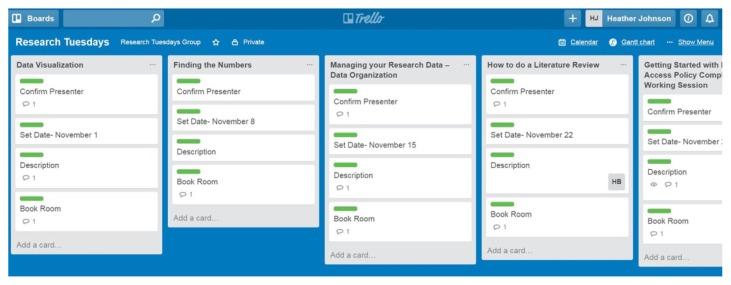
Sample Trello lists and cards

Once users have created lists, they can easily recreate that list and reorder multiple lists. For example, my library recently organized a seven-part seminar series comprising seven topics and identical task lists for each topic. To facilitate the organization of this board, we created one list and designated four cards, and then copied the list and its cards six times, changing only the list name each time. Once we assigned dates to each seminar, we reordered the lists sequentially using the platform’s drag-and-drop feature (Figure 1).

Users can easily coordinate teams and assign cards to team members (board members) by sending invitation emails directly from the project board. Once a board member has been assigned a card, the card will reflect the designation, and the board member’s initials will appear on the card. Board members do not need a Trello account to view boards, but those who create an account will benefit from greater functionality and will receive alerts pertaining to their cards. Users who work consistently with the same group of people can create teams and eliminate the need to add board members individually.

To facilitate the timely completion of tasks, users assign due dates to cards. Once users have assigned due dates, they and their board members can integrate the board into Gmail or Outlook using the Power-Ups feature. To integrate Trello boards into Outlook, users should follow these steps: Open Outlook (client or web version) → Click the calendar tab → Select “Open calendar” → Select “From Internet” → Enter the personal iCalendar feed uniform resource locator (URL) that is generated by Trello. Instructions for integrating with Gmail can be found in Trello’s FAQs. In both Gmail and Outlook, Trello calendars will appear as new calendars, which can be turned on or off as desired. Trello tasks will not appear in default user calendars.

Because Trello is a visual tool, it allows users to classify cards by assigning color-coded labels that can be further customized with words to designate statuses such as “complete” or “on hold.” For users with color blindness, Trello offers patterned rather than solid-colored labels, which can be customized with status words as well. These color-coded labels offer the most efficient way of marking tasks as complete.

Finally, Trello offers two additional standard Power-Ups: Voting and Card Aging. The Voting feature allows users to “like” a particular card, and the Card Aging feature alerts users when cards have been inactive for a period of time. As weeks pass (one, two, and four weeks), cards become increasingly transparent.

## SHORTCOMINGS

The most substantial shortcoming is that there is no straightforward way to designate tasks as complete. Currently, users may mark tasks as complete by applying a color-coded label; therefore, users would have to know that green, for example, indicates “complete.” Furthermore, to prevent a card from showing as overdue, users must delete the due date and, if desired, assign an appropriate label. While this process works fine for small projects, it would become cumbersome with larger projects.

## PRICING

Trello has three main pricing options: Free, Business Class, and Enterprise. Trello also has a semi-hidden Gold level that users unlock once they invite new team members to join the free version.

- The basic version of Trello is simply referred to as “Free.” The basic version allows users to create unlimited boards and integrate with Dropbox, Google Drive, and Box. It contains 3 basic Power-Ups (calendar integration, Voting, and Card Aging), and allows users to attach files up to 10 megabytes (MB) in size.
- Trello Gold is: $5 per month per person or free when users invite new members to join. With Gold, users can add custom backgrounds and stickers and upload files up to 250 MB in size. Users access Trello Gold from a circuitous interface; this level is not available via the traditional pricing information page.
- Business Class: Business class provides team overviews and offers integration with a number of apps, including, but not limited to, Google Hangouts, Github, Slack, and Mailchimp. Users may attach files up to 250 MB in size.
- Enterprise: Enterprise is intended for large organizations managing multiple projects and teams. Enterprise users enjoy the same features as those with Business Class with extra layers of security.

## OVERALL VALUE

For a free product, Trello is an excellent project management tool that can assist users with completing projects on time. Trello allows users to communicate with board members through using the commenting feature and by assigning members to cards. The ability to assign due dates and integrate those due dates into a personal calendar is especially useful. Furthermore, through the use of labels and due date notifications, Trello allows users to get a visual snapshot of project progress. I recommend Trello to individuals managing small to medium-sized projects and teams at the personal and professional level.

